# Approaches for handling imbalanced data used in machine learning in the healthcare field: A case study on Chagas disease database prediction

**DOI:** 10.1371/journal.pone.0320966

**Published:** 2025-05-13

**Authors:** André G. Coimbra, Cleiane G. Oliveira, Matheus P. Libório, Hasheem Mannan, Laercio I. Santos, Elisa Fusco, Marcos F.S.V. D’Angelo

**Affiliations:** 1 Graduate Program in Computer Modeling and Systems, UNIMONTES, Montes Claros, Brazil; 2 Graduate Program in Health Science, UNIMONTES, Montes Claros, Brazil; 3 University College Dublin School of Nursing, Midwifery and Health Systems, Health Sciences Centre, Belfield, Dublin 4, Ireland; 4 Instituto Federal do Norte de Minas Gerais, Montes Claros Brazil; 5 Department of Statistics, Computer Science, University of Florence, Florence, Italy; 6 Department of Computer Science, UNIMONTES, Av. Rui Braga, sn, Vila Mauricéia, Montes Claros, Brazil; National Institute of Electronics and Information Technology, INDIA

## Abstract

Machine learning has increasingly gained prominence in the healthcare sector due to its ability to address various challenges. However, a significant issue remains unresolved in this field: the handling of imbalanced data. This process is crucial for ensuring the efficiency of algorithms that utilize classification techniques, which are commonly applied in risk management, monitoring, diagnosis, and prognosis of patient health. This study conducts a comparative analysis of techniques for handling imbalanced data and evaluates their effectiveness in combination with a set of classification algorithms, specifically focusing on stroke prediction. Additionally, a new approach based on Particle Swarm Optimization (PSO) and Naive Bayes was proposed. This approach was applied to the real problem of Chagas disease. The application of these techniques aims to improve the quality of life for individuals, reduce healthcare costs, and allocate available resources more efficiently, making it a preventive action.

## Introduction

The availability of large and complex datasets from patients and medical facilities has significantly contributed to the application of machine learning methods in the healthcare field [1] [2]. These machine learning techniques can quickly analyze vast amounts of data and effectively manage complex relationships within it. As a result, they have shown potential to enhance overall health and quality of life indicators, while also enabling health specialists to advance clinical research [3].

Machine learning techniques are used in various scenarios for value prediction and decision-making support. However, for these algorithms to perform effectively, it is essential to have a substantial volume of data related to the scenarios being predicted [4]. One significant application of machine learning is in stroke prognosis. According to [5], stroke remains a global public health issue, ranking among the leading causes of death and disability in adults. [6] reports that there have been over 43 million cases since 2015, with numbers expected to rise due to an aging population. Stroke patients typically require hospitalization for treatment followed by an extended period of home-based physical, speech, and cognitive recovery [7]. Early treatment is crucial for minimizing stroke sequelae, as most risk factors are controllable [8]. While stroke diagnoses can be confirmed through clinical exams and imaging tests, these methods can be expensive and difficult to access. Machine learning techniques offer a low-cost alternative for diagnosis by mimicking human decision-making processes and rapidly analyzing large datasets.

In [9], a dataset for stroke prediction is presented, containing 43,400 patient records. However, despite the large volume of data, only 783 of these patients (1.8% of the dataset) had experienced a stroke. This situation presents a data imbalance, where the number of samples is not equally distributed between classes (patients who had a stroke and those who did not). In datasets related to medicine and public health, data imbalance must be carefully addressed, as the minority class often refers to adverse and unfavorable outcomes (such as death, disease, or high risk of hospitalization). Misclassifying a sick patient as healthy can make intervention and treatment impossible, potentially leading to severe consequences or even death.

To avoid the bias caused by data imbalance, several preprocessing techniques have been developed. These techniques involve manipulating the data used by machine learning algorithms to equalize the number of samples between classes. *Oversampling* (OS) techniques artificially create new samples so that the minority class has the same number of records as the majority class. *Undersampling* (US) techniques involve removing records from the majority class so that both classes have the same number of samples.

As previously mentioned, stroke prediction is a significant public health issue. The presence of imbalanced data can lead to inefficient machine learning models and inaccurate predictions. To address this, our work aims to conduct a detailed study in Sect of classical techniques for classifying imbalanced data and their application to healthcare problems. We present a comparison of preprocessing techniques for handling imbalanced data and analyze their impact on the efficiency of various machine learning algorithms. Stroke prediction data from [9] is used as a case study to illustrate the effectiveness of these techniques.

The main contribution of this work was the proposal of a novel approach that integrates Particle Swarm Optimization (PSO) [10] with Naive Bayes [11] to address the challenge of imbalanced data classification, detailed in Sect. By leveraging PSO, we effectively selected instances to balance the dataset, ensuring a more equitable representation of classes. Subsequently, we employed the Naive Bayes classifier for the classification task. This integrated methodology was specifically applied to predict patient mortality two years before the event occurs in an imbalanced dataset related to Chagas disease [12], demonstrating its efficacy in improving classification performance, as discussed in Sect. Our findings suggest that this approach significantly enhances the accuracy and reliability of diagnosing Chagas disease [13], offering a valuable tool for medical researchers and practitioners grappling with similar classification challenges. Conclusions drawn from this study are presented in Sect.

## Theoretical foundation

This section focuses on evaluating various supervised learning algorithms after applying data balancing techniques to stroke prediction, which is identified as a non-convex problem [9] and [14]. The supervised learning algorithms that were employed in this analysis are support vector machines (SVMs), decision trees, neural networks, k-nearest neighbors (KNN), and ensemble methods such as random forests, bagging, and AdaBoost [15, 16].

### Algorithm validation

To evaluate the efficiency of classification algorithms, the dataset is divided into training and testing sets. The algorithm is trained with the training data, and the constructed model is tested with the testing data. The results obtained with the testing set are used for comparison with other algorithms. The results are analyzed based on the predictions made by the algorithm in relation to the actual values that should have been achieved.

In this case study, the positive class is the class where a stroke occurs, and the negative class is the class where a stroke does not occur. The results are classified as True Positives (TP): the classifier correctly predicted the positive stroke cases; True Negatives (TN): the classifier correctly predicted the negative stroke cases; False Positives (FP): the classifier predicted a stroke in a patient who did not have a stroke; False Negatives (FN): the classifier identified a patient as not having a stroke when the patient actually did have a stroke.

From the results (TP, TN, FP, FN), various metrics can be calculated. The most common are accuracy, precision, and recall. Accuracy concerns how many correct values the algorithm found in relation to the entire testing set.

$$Accuracy:\frac{TP+TN}{TP+FP+TN+FN}$$ (1)

However, especially in datasets with imbalanced classes, accuracy is not a sufficient metric to evaluate the efficiency of an algorithm. The classification technique can be very efficient in predicting the majority class and poor in predicting the minority class while still having a good accuracy value.

To make a better comparison of the results, precision is also calculated. Precision is the proportion of patients who are actually positive in relation to all patients classified as positive. That is, the ratio between TP and the sum of TP with FP. The precision related to the positive class is denoted as *P*_ + _.

$$P_{+}:\frac{TP}{TP+FP}$$ (2)

We will denote *P*_−_ as the same precision calculation in relation to the negative class. That is, *P*_−_ will be the ratio between what the algorithm correctly classified as negative (TN) and the total of what the classifier claimed to be negative (the sum of TN with FN).

$$P_{-}:\frac{TN}{TN+FN}$$ (3)

Another measure of algorithm validation is recall. This measure calculates the ratio of patients classified as positive compared to the actual number of positives in the dataset. The recall related to the positive class will be denoted *R*_ + _, and concerning the negative class, *R*_−_. *R*_−_ calculates the ratio between what the algorithm correctly classified as negative (TN) in relation to the number of negative cases in the testing dataset (TN+FP).

$$R_{+}:\frac{TP}{TP+FN}$$ (4)

$$R_{-}:\frac{TN}{TN+FP}$$ (5)

One way to combine these two metrics to compare different classification algorithms is the use of the F1-Score. This metric calculates the harmonic mean between precision and recall, considering them equally in the calculation. Thus, if an algorithm has high precision and low recall, the F1-Score will return a value closer to the lower value than if the arithmetic mean were used. *F*1_ + _ calculates the F1-score for the positive class and *F*1_−_ for the negative class.

$$F1_{+}:2*\frac{P_{+}*R_{+}}{P_{+}+R_{+}}$$ (6)

$$F1_{-}:2*\frac{P_{-}*R_{-}}{P_{-}+R_{-}}$$ (7)

However, in the case study proposed in this work, the recall of the positive class becomes the most relevant metric. It is expected that the classification algorithms can predict, as accurately as possible, the positive stroke cases in the dataset and not classify sick patients as healthy, i.e., presenting the minimum number of False Negatives. One way to compare precision and recall of a classification technique, giving greater emphasis to recall, is the F2-Score (F2) metric.

$$F2:5*\frac{P_{+}*R_{+}}{(4*P_{+})+R_{+}}$$ (8)

A comparison of the AUC-ROC metric is conducted to enhance the analysis of the results. This metric measures the area under the ROC curve. Additionally, the G-mean metric is utilized to evaluate how well the algorithm balances imbalanced class data, with higher values indicating better performance. This metric is particularly useful for comparing related work.

$$G_{mean}:\sqrt{R_{+}*R_{-}}$$ (9)

### Preprocessing techniques and imbalanced data treatment

Data preprocessing is the step preceding the training of machine learning algorithms, preparing the data for a format that can be processed. To assist with this activity, there are several libraries available for scientists: Scikit-learn [4], Pandas [17] and Imbalanced-learn [18]. The initial data preprocessing techniques include handling missing values (Pandas, *fillna* feature), transforming categorical values into numerical columns (Pandas, *get_dummies* feature), transforming categorical values into binary (Scikit-learn, *LabelEncoder* feature), and normalizing data to the range between 0 and 1 (Scikit-learn, *MinMaxScaler* feature).

Specific techniques for handling imbalanced classes are Oversampling and Undersampling. Oversampling techniques address the imbalance problem by creating new samples of the underrepresented class. Undersampling techniques, on the other hand, reduce the number of samples of the majority class.

Undersampling techniques can be effective in situations where the majority class contains redundant or similar samples. However, they may also result in information loss, which can lead to biased models. Conversely, oversampling techniques can be beneficial when the dataset is small and the minority class has very few samples. Nevertheless, this approach can lead to model overfitting, as the artificially generated data may not accurately represent real-world data [19].

The Imbalanced-learn library [18] provides algorithms for both techniques. Among the Oversampling techniques are the *Random Over Sampler* and *SMOTE* algorithms. *Random Over Sampler* creates new records in the minority class by duplicating random samples with replacement. The *SMOTE* (Synthetic Minority Oversampling Technique) algorithm generates new records through interpolation. For a given sample of the minority class, *x*_*i*_, the k-nearest neighbors are identified, one of these neighbors (*x*_*zi*_) is chosen, and a new record, *x*_*new*_, is generated from the formula below, where λ is a random number between 0 and 1.

$$x_{new}=x_{i}+\lambda (x_{zi}-x_{i})$$ (10)

Among the Undersampling techniques are the *Random Under Sampler* and *NearMiss* algorithms. *Random Under Sampler* is a simple way to balance data by randomly selecting a subset of data from the majority class. *NearMiss* adds some rules for selecting samples. The rules are based on the k-nearest neighbors algorithm (KNN). Considering that it is desired to reduce the records of the negative class, the algorithm selects the negative samples whose average distance to N positive class samples is the smallest.

### Methodology

To conduct the analysis of imbalance methods, the dataset provided in [9] was used, which contains 43,400 patient records, with only 783 of them having stroke cases. This dataset is highly imbalanced since only 1.8% of the patients belong to the positive class (had a stroke), while the remaining 98.2% belong to the negative class (did not have a stroke). Each patient is described by ten characteristics, presented in Table 1 along with their respective values. The ’Stroke’ column in the dataset is the target column to be predicted, with values of 0 (no stroke) or 1 (stroke).

**Table 1:** Patient characteristics in the dataset.

Characteristics	Values
Gender	Male / Female / Other
Age	0.08 to 82
Hypertension	0 / 1
Heart disease	0 / 1
Ever married	Yes / No
Work Type	Private / Self-employed / Children / Govt job / Never worked
Residence Type	Urban / Rural
Avg glucose level	55 to 291.05
BMI	10.1 to 97.6
Smoking Status	Never smoked / Formerly Smoked / Smokes

The Python programming language was used to run the experiments, along with the Scikit-learn, Pandas, and Imbalanced-learn libraries. Google Colab was used as the programming platform, providing free access to 12.7 GB of RAM with a dual-core processor, 12 GB of RAM, and an L3 cache of 40-50 MB.

To enable data processing by the classification algorithms, initial preprocessing steps listed in Table 2 were performed. Additionally, the ’id’ column, which does not provide significant characteristics for the scenario, was removed, and the values in the ’Age’, ’avg_glucose_level’, and ’BMI’ columns were normalized to the range 0 to 1.

**Table 2:** Preprocessing steps.

Attribute	Action taken
BMI	The 1462 null values (3.4% of patients) were replaced by the median, as the values do not follow a normal distribution and, therefore, the mean would not accurately represent the data.
Gender	The 11 records with the value ’Other’ were replaced by the most frequent value ’Female’. The values ’Female’ and ’Male’ were replaced by 0 and 1 respectively.
Ever married	Replaced by 0 (No) and 1 (Yes)
Work Type	Each value was transformed into a column. When a patient has a particular value, the corresponding column is filled with 1 and the others with 0. Since there are 5 values, the dataset has 5 additional columns and the original work_type column is removed.
Residence Type	Replaced by 0 (Rural) and 1 (Urban)
Smoking Status	Since the number of null values corresponds to 30% of the patients, a new value called ’Unknown’ was created. The values were transformed into columns to retain as much information from the original dataset as possible. Since there are 4 values, 4 columns were added, and the smoking_status column was removed.

The database obtained from this initial preprocessing is referred to in the experiment as *B*_0_ and has 18 columns. From this base, others were created by applying different processing techniques for comparison by the algorithms.

- *B*_1_: *B*_0_ with the undersampling technique using the Random Under Sampler algorithm.
- *B*_2_: *B*_0_ with the undersampling technique using the NearMiss algorithm.
- *B*_3_: *B*_0_ with the oversampling technique using the Random Over Sampler algorithm.
- *B*_4_: *B*_0_ with the oversampling technique using the SMOTE algorithm.

For training the classification algorithms, the bases were divided into training (75%) and testing (25%) using the train_test_split function from the Scikit-learn package. This function performs a stratified split, meaning the class proportions of the original dataset are preserved in the training and testing sets. A random_state parameter was used consistently across all experiments to ensure the same data is in the same datasets in various experiment runs.

The chosen classification algorithms for comparison, along with their respective libraries, used to compare the preprocessing techniques were: Decision Tree - *DecisionTreeClassifier*, KNN - *KNeighborsClassifier*, SGD - *SGDClassifier*, SVM - *LinearSVC*, Neural Network - *MLPClassifier*, Random Forests - *RandomForestClassifier*, Bagging - *BaggingClassifier*, and AdaBoost - *AdaBoostClassifier*. The chosen algorithms are designed to cover a diverse range of classification techniques, including ensemble methods, to effectively capture the impact of imbalance handling techniques across various approaches. However, there are advanced variations of these algorithms such as SVM [20] and SGD that were not contemplated, being the object of future study.

The validation measures evaluated are Accuracy, *P*_ + _, *R*_ + _, *F*1_ + _, F2, AUC-ROC, G-mean *P*_−_, *R*_−_, *F*1_−_. The goal is to present the performance of the algorithms for predicting the positive class and also assess if there is a decline in the efficiency of predicting the negative class.

The tests consisted of applying the classification algorithms to all constructed bases for comparison and analyzing their efficiency, which is presented in the next section.

### Results

The first test performed consisted of applying the selected classification algorithms to the *B*_0_ dataset. Table 3 presents the results achieved. It can be observed that the algorithms achieve high accuracy values by correctly predicting the negative class even though they fail to predict any correct values for the positive class. Observing the recall and precision of the positive class (*R*_ + _ and *P*_ + _), all values are below 13%, indicating the inefficiency of these algorithms in predicting the minority class.

**Table 3:** Metrics with the $B_{0}$ dataset.

Algorithm	Accuracy	$R_{+}$	$P_{+}$	$F1_{+}$	F2	$R_{-}$	$P_{-}$	$F1_{-}$
Decision Tree	0.98	0.00	0.00	0.00	0.00	1.00	0.98	0.99
KNN	0.98	0.00	0.00	0.00	0.00	1.00	0.98	0.99
SGD	0.98	0.00	0.00	0.00	0.00	1.00	0.98	0.99
SVM	0.98	0.00	0.00	0.00	0.00	1.00	0.98	0.99
Neural Network	0.98	0.00	0.00	0.00	0.00	1.00	0.98	0.99
Random Forest	0.98	0.00	0.12	0.01	0.00	1.00	0.98	0.99
Bagging	0.98	0.04	0.13	0.06	0.05	0.99	0.98	0.99
Adaboost	0.98	0.00	0.00	0.00	0.00	1.00	0.98	0.99

Table 4 presents the accuracy values achieved by the algorithms on each constructed dataset. The results show that with undersampling techniques (datasets *B*_1_ and *B*_2_), there is a decrease in the accuracy of the algorithms, whereas with oversampling techniques, some results are similar to the *B*_0_ dataset as seen with the KNN, RandomForest, and Bagging algorithms. However, the accuracy measure is not sufficient to demonstrate if the algorithms are effective in predicting the positive class. As observed in Table 3, most algorithms predict the majority class (negative) very well to the detriment of the minority class (positive).

**Table 4:** Accuracy comparison.

Algorithm	$B_{0}$	$B_{1}$	$B_{2}$	$B_{3}$	$B_{4}$
Decision Tree	0.98	0.78	0.83	0.78	0.80
KNN	0.98	0.72	0.79	0.98	0.92
SGD	0.98	0.79	0.81	0.77	0.79
SVM	0.98	0.78	0.79	0.78	0.79
Neural Network	0.98	0.77	0.81	0.87	0.86
Random Forest	0.98	0.73	0.81	1.00	0.95
Bagging	0.98	0.75	0.82	0.99	0.98
AdaBoost	0.98	0.79	0.80	0.81	0.88

To evaluate the performance of the classification techniques for predicting the positive class, the data presented in Tables 5, 6 and 7 are used. Table 5 presents the recall and precision values for the positive class in each constructed dataset. The data shows that the performance of the algorithms increased considerably compared to the *B*_0_ dataset where no technique was applied to address the imbalance.

**Table 5:** Comparison of recall and precision for the positive class.

Algorithm	$B_{0}$		$B_{1}$		$B_{2}$		$B_{3}$		$B_{4}$	
	*R*_ + _	*P*_ + _	*R*_ + _	*P*_ + _	*R*_ + _	*P*_ + _	*R*_ + _	*P*_ + _	*R*_ + _	*P*_ + _
Decision Tree	0.00	0.00	0.80	0.75	0.88	0.78	0.76	0.83	0.77	0.86
KNN	0.00	0.00	0.73	0.73	0.91	0.67	0.96	1.00	0.88	0.98
SGD	0.00	0.00	0.64	0.70	0.68	0.63	0.68	0.67	0.77	0.75
SVM	0.00	0.00	0.68	0.66	0.70	0.62	0.75	0.75	0.77	0.76
Neural Network	0.00	0.00	0.71	0.71	0.76	0.76	0.94	0.83	0.93	0.88
Random Forest	0.00	0.12	0.72	0.68	0.91	0.82	0.97	0.99	0.93	0.97
Bagging	0.04	0.13	0.71	0.71	0.90	0.80	1.00	0.97	0.97	0.98
AdaBoost	0.00	0.00	0.71	0.64	0.68	0.58	0.85	0.77	0.82	0.82


**Table 6:** Comparison of $F1_{+}$ and *F*2 in the positive class.

Algorithm	$B_{0}$		$B_{1}$		$B_{2}$		$B_{3}$		$B_{4}$	
	*F*1_ + _	*F*2	*F*1_ + _	*F*2	*F*1_ + _	*F*2	*F*1_ + _	*F*2	*F*1_ + _	*F*2
Decision Tree	0.00	0.00	0.77	0.79	0.82	0.86	0.79	0.77	0.81	0.79
KNN	0.00	0.00	0.73	0.73	0.77	0.85	0.98	0.97	0.93	0.90
SGD	0.00	0.00	0.80	0.78	0.80	0.85	0.79	0.76	0.80	0.78
SVM	0.00	0.00	0.79	0.78	0.78	0.82	0.79	0.76	0.80	0.77
Neural Network	0.00	0.00	0.78	0.77	0.80	0.84	0.87	0.85	0.87	0.84
Random Forest	0.01	0.00	0.72	0.74	0.81	0.83	1.00	1.00	0.95	0.94
Bagging	0.06	0.05	0.76	0.75	0.82	0.83	0.99	0.99	0.98	0.98
AdaBoost	0.00	0.00	0.79	0.80	0.80	0.81	0.82	0.80	0.88	0.88


**Table 7:** AUC-ROC comparison.

Algorithm	$B_{0}$	$B_{1}$	$B_{2}$	$B_{3}$	$B_{4}$
Decision Tree	0.50	0.78	0.83	0.78	0.80
KNN	0.50	0.72	0.80	0.98	0.92
SGD	0.50	0.79	0.81	0.77	0.79
SVM	0.50	0.78	0.79	0.78	0.79
Neural Network	0.50	0.77	0.81	0.86	0.86
Random Forest	0.50	0.73	0.81	1.00	0.95
Bagging	0.52	0.75	0.82	1.00	0.98
AdaBoost	0.50	0.79	0.80	0.81	0.88

In Table 6, the *F*1_ + _ and *F*2 metrics for the positive class are presented, comparing recall and precision for each applied technique. The *F*1_ + _ metric gives equal importance to both metrics, while *F*2 considers recall twice as much as precision. The results reinforce that imbalance treatment techniques considerably improve the efficiency of the algorithms. Table 7 presents the AUC-ROC values for comparison. These values are crucial for evaluating the performance of different models, as they provide insight into the models ability to distinguish between classes. By examining these metrics, we can better understand how effectively each model handles imbalanced data and make more informed decisions about which approach may be most suitable for a given application.

**Table 8:** Comparison of recall and precision for the negative class.

Algorithm	$B_{0}$		$B_{1}$		$B_{2}$		$B_{3}$		$B_{4}$	
	*R*_−_	*P*_−_	*R*_−_	*P*_−_	*R*_−_	*P*_−_	*R*_−_	*P*_−_	*R*_−_	*P*_−_
Árvore de decisão	1.00	0.98	0.75	0.81	0.79	0.88	0.82	0.73	0.84	0.74
KNN	1.00	0.98	0.72	0.72	0.73	0.93	1.00	0.96	0.98	0.87
SGD	1.00	0.98	0.81	0.73	0.76	0.89	0.81	0.72	0.83	0.73
SVM	1.00	0.98	0.79	0.75	0.75	0.86	0.80	0.73	0.82	0.73
Rede Neural	1.00	0.98	0.78	0.74	0.76	0.88	0.91	0.81	0.90	0.81
Random Forest	1.00	0.98	0.70	0.77	0.79	0.85	1.00	1.00	0.96	0.93
Bagging	0.99	0.98	0.75	0.74	0.81	0.82	1.00	0.99	0.98	0.98
AdaBoost	1.00	0.98	0.78	0.79	0.77	0.83	0.84	0.77	0.89	0.87


Although the recall for the positive class is the most important in stroke prognosis, the performance of the algorithms in the negative class should also be analyzed. Table 9 presents the recall and precision values obtained by the algorithms in the study databases. It can be observed that most results decrease compared to the *B*_0_ base but always remain above 70%.

**Table 9:** Comparison of recall (R) and precision (P) in the negative class.

Algorithm	$B_{0}$		$B_{1}$		$B_{2}$		$B_{3}$		$B_{4}$	
	R	P	R	P	R	P	R	P	R	P
Decision Tree	0.99	0.97	0.71	0.79	0.83	0.85	0.76	0.80	0.76	0.81
KNN	0.99	0.94	0.79	0.74	0.81	0.83	0.82	0.79	0.76	0.82
SGD	0.99	0.95	0.75	0.78	0.84	0.87	0.77	0.82	0.78	0.82
SVM	0.99	0.95	0.76	0.80	0.84	0.87	0.81	0.82	0.80	0.83
Neural Network	0.99	0.94	0.79	0.81	0.85	0.89	0.84	0.85	0.85	0.86
Random Forest	0.99	0.95	0.75	0.79	0.85	0.86	0.99	0.98	0.95	0.95
Bagging	0.99	0.92	0.73	0.75	0.84	0.88	0.87	0.89	0.88	0.87
AdaBoost	0.99	0.96	0.79	0.83	0.85	0.89	0.84	0.86	0.85	0.88


As in the results for the positive class, it can be seen that the efficiency of the algorithms is significantly improved with imbalance treatment techniques, despite a slight decrease in most cases.

In Table 10, the results for the *F*1_−_ measure are presented. As the negative class is not the main issue in predicting stroke prognosis, it was considered sufficient to evaluate precision and recall for this class without the need to calculate the *F*2 metric. The results show that even by reducing the data of the negative class or artificially creating data for the positive class, the algorithms still achieve good results (above 70%) in predicting the prognosis for those who do not have a stroke. The classification algorithms used for comparison, along with their respective scikit-learn libraries [4], used to compare the preprocessing techniques were: Decision Tree - *DecisionTreeClassifier*, KNN - *KNeighborsClassifier*, SGD - *SGDClassifier*, SVM - *LinearSVC*, Neural Network - *MLPClassifier*, Random Forests - *RandomForestClassifier*, Bagging - *BaggingClassifier*, and AdaBoost - *AdaBoostClassifier*. The chosen algorithms are designed to cover a diverse range of classification techniques, including ensemble methods, to effectively capture the impact of imbalance handling techniques across multiple approaches.

**Table 10:** Comparison of $F1_{1}$ in the negative class.

Algorithm	$B_{0}$	$B_{1}$	$B_{2}$	$B_{3}$	$B_{4}$
Decision Tree	0.99	0.78	0.83	0.79	0.79
KNN	0.99	0.72	0.81	0.98	0.92
SGD	0.99	0.77	0.82	0.79	0.78
SVM	0.99	0.77	0.80	0.79	0.78
Neural Network	0.99	0.76	0.82	0.87	0.86
Random Forest	0.99	0.73	0.82	1.00	0.94
Bagging	0.99	0.74	0.81	0.99	0.98
AdaBoost	0.99	0.78	0.80	0.82	0.88

With imbalance treatment techniques, the algorithms were able to predict (recall) between 73% (KNN) and 100% (RandomForest) of patients who indeed have a positive prognosis for stroke, given the study’s database. Comparing the techniques, a slightly higher efficiency gain is observed among the Oversampling techniques compared to the Undersampling techniques. It is believed that this difference is due to the larger amount of available information, allowing the algorithms to identify more patterns in the data.

The reference work, [9], presents a hybrid proposal for handling data imbalance in the same stroke database. The metrics presented here in common with this study are Accuracy, *R*_−_, and *R*_ + _. For comparison, the values obtained in [9] and the values obtained in the previous tables are placed side by side in Table 11. For each analyzed base, the result achieved by the algorithm that obtained the best *F*2 value is presented: *B*_0_ - Bagging, *B*_1_ - AdaBoost, *B*_2_ - Decision Tree, *B*_3_ - Random Forest, and *B*_4_ - Bagging. The techniques studied present better results compared to the reference work, reinforcing the increased efficiency of the algorithms with the presented imbalance treatment techniques.

**Table 11:** Comparison of the results achieved by the algorithm with the best *F*2 score against the results presented in [9].

Data Base		$B_{0}$	$B_{1}$	$B_{2}$	$B_{3}$	$B_{4}$
Algorithm	[9]	Bagging	AdaBoost	Decision Tree	Random Forest	Bagging
Accuracy	0.72	0.98	0.79	0.83	1.00	0.98
*R*_ + _	0.67	0.04	0.80	0.88	1.00	0.98
*R*_−_	0.33	0.99	0.78	0.79	1.00	0.98
*G*_*mean*_	0.47	0.20	0.79	0.83	1.00	0.98

### Critical analysis of results

This section systematically evaluates the efficacy of oversampling and undersampling techniques in addressing the challenge of imbalanced data within machine learning applications for stroke prediction in healthcare. Specifically, oversampling methods, such as the Synthetic Minority Over-sampling Technique (SMOTE), and undersampling strategies, like the NearMiss algorithm, were applied to a dataset characterized by a significant imbalance in stroke incidence among patients.

Our analysis indicates that the judicious application of these techniques can substantially enhance the performance of classification algorithms in predicting stroke events. Notably, oversampling demonstrated a slight advantage over undersampling, likely due to the increased data volume providing additional learning opportunities for the algorithms. This improvement is crucial in healthcare settings, where accurate risk assessment and timely intervention are vital for stroke management and patient outcomes.

The findings of this study underscore the necessity of employing appropriate data balancing techniques to ensure the reliability and effectiveness of machine learning models in medical applications. Further research is encouraged to explore additional data balancing methodologies and their impact on other imbalanced healthcare datasets, with the goal of advancing the field of operational research and machine learning in healthcare. Building on this, we propose an alternative methodology presented in Sect, comparing it with the results discussed here, and applying it to a death prediction problem in a Chagas disease database, as detailed in Sect.

## A novel approach utilizing PSO and Naive Bayes for imbalanced data handling

This section describes the contribution of a novel approach to classifying imbalanced data using the Particle Swarm Optimization (PSO) algorithm to select the best instances, variables, and parameters in building a classification model. Previous studies have explored the use of Particle Swarm Optimization (PSO) to handle imbalanced data, though often focusing on isolated aspects. For instance, [21] proposed a method for classifying imbalanced data using PSO to optimize a neural network. This approach enhanced classification performance by penalizing errors from the minority class and adjusting the importance of variables. Conversely, [22] suggested a combination of PSO with the Naive Bayes (NB) classifier to select the best training data, thereby improving model efficiency. However, the proposed work simultaneously performs instance selection, variable selection, and parameter tuning. The main goal is to achieve higher accuracy and generalization, assessing the F1 Score for each class, while reducing model complexity through adaptive variable selection.

The combination of Particle Swarm Optimization (PSO) with Naive Bayes (NB) is particularly promising because it harnesses the complementary strengths of each technique. PSO is adept at exploring the solution space by efficiently adjusting parameters. Meanwhile, NB is a probabilistic classifier that assumes conditional independence among variables and is notable for its computational efficiency, especially with large datasets. However, as noted by [23], NB can lead to inaccurate estimates when the independence assumptions are not met. The synergy of these two approaches is powerful because PSO can optimize the parameters of NB, enhancing classification accuracy by finding more suitable configurations. Thus, this combination improves the model’s ability to handle real-world data, where variables are often correlated.

The study unfolds in two main stages. The first stage involves data normalization, addressed in Sect, crucial in preprocessing, especially when working with machine learning algorithms sensitive to variable scales. The second stage, detailed in Sect, deals with configuring the PSO algorithm and outlines the development of the objective function. This function evaluates the quality of solutions obtained by PSO, which selects instances and variables based on established thresholds, trains a Naive Bayes model with the selected data, and computes the weighted F1 Score of predictions. The function also incorporates adaptive penalization to balance model complexity.

### Data standardization

Data standardization is a widely used technique in statistics and machine learning to transform different variables into a common scale, facilitating comparison and analysis of the data. One of the most common methods of standardization is using the *z-score* [24].

The *z-score*, also known as the standardized score, is a measure that describes the position of a value relative to the mean of a dataset, in units of standard deviation. The *z-score* of a value *x* is calculated using the following formula:

$$z=\frac{x-\mu }{\sigma }$$ (11)

where:

- *x* is the value being standardized.
- μ is the mean of the dataset.
- σ is the standard deviation of the dataset.

The *z-score* indicates how many standard deviations a value is above or below the mean. A positive *z-score* indicates that the value is above the mean, while a negative *z-score* indicates that the value is below the mean. For example:

- A *z-score* of 0 means that the value is equal to the mean.
- A *z-score* of 1 means that the value is one standard deviation above the mean.
- A *z-score* of -1 means that the value is one standard deviation below the mean.

Standardizing data using the *z-score* is particularly useful when comparing data that are on different scales or units. By converting the data to *z-scores*, all variables will have a mean of zero and a standard deviation of one, making them directly comparable.

The *z-score* is a powerful tool for data standardization, allowing variables of different scales to be compared uniformly. Its application is essential in various fields of statistics and machine learning, where accurate comparison of data is crucial.

### Particle swarm optimization (PSO)

PSO is a population-based optimization algorithm. Each particle, initialized randomly, represents a potential solution and moves through the search space influenced by its best-found position and the best-found position of its neighbors [25].

#### Parameters.

The definition of parameters is of utmost relevance due to their impact on the strategy of exploring possible solutions.

In this work, the criterion used to select variables and instances from the potential solution provided by PSO was the configuration of a selection threshold. That is, the variable or instance is selected when the suggestion, within the bounds of the search space, exceeds the predefined threshold.

Weight Inertia was updated using the cooling method at each iteration, the cognitive component was progressively increased at each iteration while the social component remained static. To converge rapidly and efficiently, global neighborhood exploration was adopted, where each particle can communicate directly with all others influenced by the best result found by any particle in the swarm.

#### Objective function.

The objective function plays a central role in the optimization process and is responsible for evaluating, at each iteration, the quality of each candidate solution generated by the Particle Swarm Optimization (PSO) algorithm — that is, a specific combination of selected variables and instances. In this work, the objective function was defined with the following steps:

- Definition of training data from the selection of variables and instances based on the configured threshold.
- Execution of the Naive Bayes classification model with the selected instances, variables, and smoothing parameter.
- Prediction using the trained model on the test set.
- Calculation of the weighted F1 Score of the classes.
- Application of penalization to the F1 Score based on the number of selected variables to control the model’s complexity, as described in the following formula:

$$F_{objective}=\left(F1C_{0}\cdot p_{1}\right)+\left(F1C_{1}\cdot p_{2}\right)-P\cdot N^{2}$$ (12)

where:

- *F*1*C*_0_ is the F1 Score for class 0.
- *F*1*C*_1_ is the F1 Score for class 1.
- *p*_1_ and *p*_2_ are the weights assigned to each class.
- *P* is the penalization factor.
- *N* is the number of selected variables.

### Comparison with the presented results

In this section, we aim to provide a detailed comparison of our findings with the results previously presented in Table 11 of Sect. By analyzing various metrics, we can better understand the performance and reliability of our approach in identifying the target class. Specifically, we focus on the accuracy, *R*_−_, and *R*_ + _ values to highlight their respective behaviors and effectiveness. This comparison will help us draw meaningful conclusions about the robustness of our methodology and identify the most reliable metric for future analyses.

Accuracy and the *R*_−_ value exhibited behavior similar to the results shown in Table 11. This consistency indicates reliability in the data obtained for these metrics. However, it is important to highlight that the *R*_ + _ value surpassed all other results. This superior performance of *R*_ + _ suggests that this metric is the most effective and accurate for identifying the target class. Therefore, we can conclude that among all the metrics analyzed, *R*_ + _ offers the best performance in correctly identifying the target class, reinforcing its importance and usefulness in future analyses.

## Applications in various imbalanced datasets

To illustrate the robustness and effectiveness of the proposed method, it was applied to three distinct imbalanced datasets: Churn prediction [26], Diabetes [28], and Lung cancer [27]. These datasets were selected to cover a variety of scenarios and challenges typically encountered in imbalanced classification problems. The results presented in Table 12 compare the average performance across 10 runs in each datasets of the proposed method (PSO+NB) with classification performed by Naive Bayes algorithm, multilayer perceptron (MLP) neural network and Genetic Algorithm combined with Naive Bayes (GA+NB) using appropriate performance metrics for imbalanced classification problems, such as the F1-Score, F2-Score, and AUC-ROC, providing a comprehensive view of the method’s effectiveness in various contexts. This allowed for a rigorous evaluation of the proposed method’s ability to handle different types of imbalances and its competitiveness compared to the most advanced techniques currently available.

**Table 12:** Comparison of average performance across 10 runs on different datasets.

Dataset	Method	Acc	R+	P+	F1+	F2	R-	P-	F1-	AUC-ROC
Churn	Naive Bayes	0.75	0.73	0.52	0.60	0.67	0.75	0.88	0.81	0.74
	MLP	0.76	0.50	0.55	0.52	0.51	0.85	0.83	0.84	0.68
	GA+NB	0.78	0.70	0.57	0.63	0.67	0.81	0.88	0.84	0.75
	PSO+NB	0.80	0.72	0.60	0.65	0.69	0.82	0.89	0.86	0.77
Diabetes	Naive Bayes	0.74	0.58	0.64	0.61	0.59	0.82	0.78	0.80	0.70
	MLP	0.75	0.59	0.67	0.63	0.60	0.84	0.79	0.82	0.72
	GA+NB	0.79	0.63	0.74	0.68	0.65	0.88	0.81	0.85	0.75
	PSO+NB	0.84	0.71	0.81	0.75	0.73	0.90	0.86	0.88	0.81
Lung Cancer	Naive Bayes	0.90	0.95	0.94	0.94	0.95	0.55	0.65	0.58	0.75
	MLP	0.89	0.95	0.92	0.94	0.95	0.46	0.62	0.52	0.71
	GA+NB	0.94	0.97	0.96	0.97	0.97	0.75	0.79	0.77	0.86
	PSO+NB	0.97	0.98	0.98	0.98	0.98	0.88	0.88	0.88	0.93


## Application to an imbalanced Chagas disease database

Chagas Disease (CD) is recognized by the World Health Organization as a neglected tropical disease, and despite partial control, it remains a public health problem. Although many patients remain in the indeterminate clinical form of the disease, it is estimated that 30% may develop cardiac abnormalities that can lead to patient death. It is estimated that in Latin America alone, there are about 5.7 million people infected and an annual mortality rate of 12,000 cases [13].

The study conducted in [29] estimates that 80% of people infected with CD will not have access to adequate diagnosis and treatment, resulting in a high mortality rate and significant social costs. In this context, Machine Learning has proven to be a promising strategy as it can define interventions to reduce the impact of CD [13, 30]. Furthermore, adopting a tool capable of predicting the risk of death in advance for a person with CD can assist healthcare professionals in managing the disease, especially in regions with limited access to complex examinations.

In this study, we apply the proposed methodology to a dataset on CD, as described in [13] and made available in [12]. The objective was to predict patient mortality two years before the event occurs. The dataset comprises interview variables and supplementary exam variables, totaling 128 predictor variables, as well as the class ’death’ or ’non-death’ in two years (predicted variable). The data is sourced from the SaMi-Trop Cohort, which includes 551 CD patients recruited from 21 municipalities in the state of Minas Gerais (Brazil). Of the 551 participants, 134 (24.32%) died within 2 years, indicating a considerable class imbalance.

Predictor values were collected in 2013 and 2014, and the outcome (death) was collected in 2015 and 2016. For data collection, visits were conducted at primary healthcare units in the municipalities, where participants were interviewed, had blood samples collected, and electrocardiogram (ECG) exams performed. The interview covered sociodemographic issues, lifestyle habits, clinical history, disease treatment, physical activity, and quality of life.

For model validation, in each execution, the dataset was divided in a stratified manner, with 80% for training and 20% for testing. Stratified splitting ensures class proportionality. In each execution, the training and testing split was performed randomly to obtain different data partitions in each run, mitigating specific sample biases and ensuring that the model’s performance is not attributed to a single partition. Additionally, to ensure that the variables in the dataset are on the same scale, data normalization was performed using the Z-score technique, which enhances the algorithm’s effectiveness.

To establish a baseline for comparison, classification runs were performed exclusively using the Naive Bayes algorithm with the dataset. The results of this execution are presented in Table 13. In all runs, low overall accuracy and class precisions were observed, reflecting the imbalance of 75.68% in class 0 (not dead) and 24.32% in class 1 (dead).

**Table 13:** Naive Bayes classification.

Execution	Acc	R+	P+	F1+	F2	R-	P-	F1-	AUC-ROC	Variables	Train Instances
1	0.31	0.93	0.25	0.39	0.60	0.11	0.82	0.19	0.52	128	440
2	0.42	1.00	0.30	0.46	0.68	0.24	1.00	0.38	0.62	128	440
3	0.34	0.81	0.24	0.38	0.56	0.19	0.76	0.30	0.50	128	440
4	0.66	0.93	0.41	0.57	0.74	0.57	0.96	0.72	0.75	128	440
5	0.37	0.89	0.26	0.41	0.60	0.20	0.85	0.33	0.55	128	440
6	0.41	1.00	0.29	0.45	0.68	0.23	1.00	0.37	0.61	128	440
7	0.41	0.96	0.29	0.44	0.65	0.23	0.95	0.37	0.59	128	440
8	0.66	0.74	0.39	0.51	0.63	0.63	0.88	0.74	0.69	128	440
9	0.43	0.93	0.29	0.44	0.64	0.27	0.92	0.42	0.60	128	440
10	0.28	0.93	0.24	0.38	0.59	0.07	0.75	0.13	0.50	128	440

To expand the bases for comparison and enhance the robustness of the method, tests were also conducted using the Genetic Algorithm (GA) combined with Naive Bayes on the same dataset. The Genetic Algorithm, which optimizes through the processes of selection (Roulette Wheel Selection), crossover (Single-Point Crossover), and mutation (Bitflip Mutation) of candidate solutions, was executed with the same objective function used in the PSO+NB method, to select the set of variables and instances aimed at improving the performance of the NB classifier. The results of these executions showed a noticeable improvement compared to using NB alone and can be seen in Table 15.

**Table 14:** GA parameters and objective function.

Description	Value
Penalty (*P*)	7.81E-03
Weight (*p*_1_ and *p*_2_)	0.5
Population	50
Generations	200
Mutation rate	0.01
Initialization	Random

**Table 15:** GA optimization + Naive Bayes classification.

Execution	Acc	R+	P+	F1+	F2	R-	P-	F1-	AUC-ROC	Variables	Train Instances
1	0.79	0.70	0.56	0.62	0.67	0.82	0.90	0.86	0.76	44	206
2	0.72	0.59	0.44	0.51	0.56	0.76	0.85	0.81	0.68	41	198
3	0.75	0.33	0.47	0.39	0.35	0.88	0.80	0.84	0.61	40	222
4	0.68	0.56	0.39	0.46	0.51	0.73	0.84	0.78	0.64	40	214
5	0.68	0.70	0.41	0.52	0.62	0.68	0.88	0.77	0.69	43	217
6	0.69	0.67	0.42	0.51	0.60	0.70	0.87	0.78	0.68	42	218
7	0.77	0.81	0.52	0.64	0.73	0.76	0.93	0.84	0.79	43	224
8	0.73	0.63	0.46	0.53	0.59	0.76	0.86	0.81	0.70	40	227
9	0.68	0.78	0.42	0.55	0.66	0.65	0.90	0.76	0.72	44	226
10	0.77	0.81	0.51	0.63	0.73	0.75	0.93	0.83	0.78	47	232

Table 14 describes the parameters used in the model GA optimization configuration. The configuration variable *Penalty* (*P*) was calculated as the inverse of the total number of predictive variables in the model. This way, selecting a larger number of variables incurs a higher penalty, promoting a reduction in model complexity. The *Weight* (*p*_1_ and *p*_2_) was assigned to ensure that the F1 Score for both classes is the same. The *Mutation rate* was chosen empirically, while the *Population* and *Generations* variables were assigned values equivalent to those tested in the PSO+NB model to allow for comparability.

From the perspective of comparing methods, classification tests were also conducted using a multilayer perceptron (MLP) neural network on the same dataset. The architecture consisted of two hidden layers, configured with the parameters described in Table 16. The results obtained showed a significant improvement compared to using NB alone, as seen in Table 17.

**Table 16:** MLP parameters.

Description	Value
Hidden Layer	128, 64
Activation	ReLU
Optmizer	Adam
Learning Rate	0.001
Max. Iterations	200

**Table 17:** MLP classification.

Execution	Acc	R+	P+	F1+	F2	R-	P-	F1-	AUC-ROC	Variables	Train Instances
1	0.79	0.52	0.58	0.55	0.53	0.88	0.85	0.87	0.70	128	440
2	0.80	0.56	0.60	0.58	0.56	0.88	0.86	0.87	0.72	128	440
3	0.80	0.56	0.60	0.58	0.56	0.88	0.86	0.87	0.72	128	440
4	0.85	0.52	0.78	0.62	0.56	0.95	0.86	0.90	0.74	128	440
5	0.85	0.63	0.71	0.67	0.64	0.92	0.89	0.90	0.77	128	440
6	0.79	0.44	0.60	0.51	0.47	0.90	0.84	0.87	0.67	128	440
7	0.81	0.59	0.62	0.60	0.60	0.88	0.87	0.88	0.74	128	440
8	0.82	0.70	0.61	0.66	0.68	0.86	0.90	0.88	0.78	128	440
9	0.83	0.59	0.67	0.63	0.61	0.90	0.87	0.89	0.75	128	440
10	0.82	0.63	0.63	0.63	0.63	0.88	0.88	0.88	0.76	128	440

Table 18 describes the parameters used in the model PSO optimization configuration. The *Interval search of Variables and Instances* was defined based on the nature of the problem, where the objective is to decide whether to select a particular variable or instance. The *Interval search of Smoothing NB* was determined empirically. The value of *Variable threshold* was set to increase the likelihood of selecting the fewest possible variables, thus reducing model complexity, which is further enhanced by the configuration variable Penalty (*P*). This penalty was calculated as the inverse of the total number of predictive variables, meaning that selecting more variables incurs a higher penalty. The *Instance threshold* was configured to balance the probability of selecting an instance. The Weight (*p*_1_ and *p*_2_) was assigned to ensure that the F1 Score of both classes is the same. The *Weight Inertia* (*w*) was updated in each execution using the interval tested in the study [31]. For the *Cognitive* (*c*_1_) and *Social* (*c*_2_) variables, the value 2.0 suggested in [32] was observed; however, for each execution, *c*_1_ was updated linearly, gradually increasing the emphasis on the individual. The *Velocity* variable was set to half the search space for controlled exploration. The variables *Number of particles* and *Iterations* were chosen empirically.

**Table 18:** PSO parameters and objective function.

Description	Value
Interval search of Variables and Instances	[0, 1]
Interval search of Smoothing NB	[1, 1e-9]
Variable threshold	0.7
Instance threshold	0.5
Penalty (*P*)	7.81E-03
Weight (*p*_1_ and *p*_2_)	0.5
Weight Inertia (*w*)	0.9 to 0.4
Cognitive (*c*_1_)	1.6 to 2.0
Social (*c*_2_)	2.0
Number of particles	50
Velocity	-0.5 to 0.5
Iterations	200
Initialization	Random
Neighborhood exploration	Global

The results obtained in the execution of the PSO+NB model showed a substantial gain, both in overall performance and in individual class performance, as observed in Table 19. In this context, it is worth highlighting that the average AUC-ROC was 0.59 for the NB executions, 0.70 for the GA+NB, 0.73 for the MLP, and 0.81 for the PSO+NB. The results clearly show that the PSO+NB model approach provided significant improvements in overall accuracy and in precision, recall, AUC-ROC and F1 metrics for both classes. The optimized classification model managed to find a set of variables and tests capable of handling the data imbalance, resulting in a more balanced classification. These results surpass those presented by [13], indicating that the proposed methodology can be applied to various other problems in the health field that suffer from class imbalance issues.

**Table 19:** PSO optimization + Naive Bayes classification.

Execution	Acc	R+	P+	F1+	F2	R-	P-	F1-	AUC-ROC	Variables	Trian Instances
1	0.85	0.56	0.75	0.64	0.59	0.94	0.87	0.90	0.75	1	226
2	0.85	0.85	0.64	0.73	0.80	0.84	0.95	0.89	0.85	2	240
3	0.87	0.81	0.71	0.76	0.79	0.89	0.94	0.91	0.85	2	220
4	0.82	0.63	0.63	0.63	0.63	0.88	0.88	0.88	0.76	2	218
5	0.82	0.96	0.58	0.72	0.85	0.77	0.98	0.87	0.87	2	215
6	0.90	0.85	0.77	0.81	0.83	0.92	0.95	0.93	0.88	3	209
7	0.85	0.59	0.73	0.65	0.62	0.93	0.88	0.90	0.76	2	235
8	0.81	0.85	0.58	0.69	0.78	0.80	0.94	0.86	0.82	2	219
9	0.80	0.70	0.58	0.63	0.67	0.83	0.90	0.86	0.77	2	227
10	0.85	0.56	0.75	0.64	0.59	0.94	0.87	0.90	0.75	1	225

## Conclusion

In conclusion, this paper presents a comprehensive comparison of preprocessing techniques for handling imbalanced data in predicting stroke, evaluating their impact on the efficiency of various machine learning algorithms. Moreover, we integrated Particle Swarm Optimization (PSO) with Naive Bayes to predict patient mortality up to two years before the event in an imbalanced dataset related to Chagas disease. Our findings indicate that this approach significantly enhances the accuracy and reliability of diagnosing Chagas disease, offering a valuable tool for medical researchers and practitioners addressing the challenge of imbalanced data. In real-world healthcare scenarios, false positives could lead to unnecessary anxiety for patients and unwarranted tests and treatments, while false negatives might result in missed diagnoses and delayed critical interventions. By effectively handling imbalanced data, the methodology employed in this paper enables the prediction of mortality risk for individuals with rare health conditions, assisting healthcare professionals in managing such diseases, particularly in regions with limited access to complex diagnostic procedures. Addressing these real-world implications underscores the practical significance of our approach in improving patient outcomes and optimizing healthcare resources.
